# A Rare Presentation of Peritoneal Tuberculosis Mimicking Malignancy

**DOI:** 10.1177/2324709616679191

**Published:** 2016-11-22

**Authors:** Thein Swe, Akari Thein Naing, Zaw Win Phyo, Malar Thwin

**Affiliations:** 1 Department of Internal Medicine, Interfaith Medical Center, New York, NY, USA

**Keywords:** peritoneal tuberculosis, serum cancer antigen 125, rheumatoid factor

## Abstract

Our search of literature revealed combined elevations of serum cancer antigen 125 levels and rheumatoid factor levels in a patient with peritoneal tuberculosis has rarely been reported. Thus, we describe the case of a 63-year-old female with large abdominal ascites and malignancy was ruled out with biopsy. High levels of serum cancer antigen and rheumatoid factor were noted. Physicians should be aware that tuberculosis infection could induce elevation of rheumatoid factor levels in the absence of rheumatologic symptoms or disease. A high index of suspicion is required because peritoneal tuberculosis is a great mimicker of other abdominal pathology, especially intraabdominal malignancies and can mislead physicians to undergo unnecessary interventions.

## Introduction

Cancer antigen (CA 125) is a useful tumor marker for evaluation of ovarian cancer. It can also be elevated in pelvic inflammatory disease, pregnancy, peritonitis, and leiomyoma of the uterus.^1^ Tuberculosis (TB) is a protean disease that can present with diverse symptoms and sometimes mimicking malignancy especially in postmenopausal women who present with ascites, weight loss, and high CA 125 levels. Therefore, it is of paramount importance to consider peritoneal TB as one of the causes of elevation of CA 125 for patients who immigrated from countries with a high prevalence of TB and after malignancy has been ruled out.^2^

Tuberculosis may induce to increase a variety of autoimmune markers such as antinuclear antibody, anticardiolipin, antineutrophil cytoplasmic antibodies, and rheumatoid factor.^3^ High titers of rheumatoid factor are more common in pulmonary TB and tuberculous arthritis compared to peritoneal TB. Our patient was diagnosed with peritoneal TB, and laboratory tests revealed high CA 125 and rheumatoid factor levels.

## Case Presentation

A 63-year-old female with past medical history of diabetes mellitus and hypertension presented to emergency department for abdominal distention for 2 weeks associated with poor appetite and weight loss. She denied current fever, chills, rigors, night sweat, cough, abdominal pain, leg edema, facial swelling, bowel problems, headache, vomiting, nausea, dizziness, blurry vision, chest pain, palpitations, paroxysmal nocturnal dyspnea, orthopnea, or similar illness in the past. The patient does not have any family history of rheumatologic diseases, and she denied any rash, joint pain, morning stiffness, and muscle weakness. She is a nonsmoker. She lives in central Africa and travelled to the United States to visit her daughter.

Initial vital signs included temperature 97°F (36.1°C), pulse rate 103 beats/minute, respiratory rate 18 breaths/minute, blood pressure 137/97 mm Hg, and oxygen saturation 96% on room air. Physical examination showed markedly distended abdomen, soft, nontender without palpable mass. There was fluid thrill with shifting dullness. Bowel sounds were normoactive. Respiratory exam was unremarkable except for mildly reduced air entry in lung bases.

Laboratory tests showed white blood cells 6.4 × 10^9^/L, hemoglobin 11 g/dL, platelet counts 367 000/µL, bilirubin 0.5 mg/dL, aspartate transaminase 36 IU/L, alanine transaminase 22 IU/L, alkaline phosphatase 67 IU/L, and albumin 2.9 g/dL. Tumor marker α-fetoprotein level, carcinoembryonic antigen, cancer antigen 19-9, coagulation profile, lipase, hepatitis A, B, and C panels, B-type natriuretic peptide, antinuclear antibody, kidney function tests, and thyroid function tests were within normal limits. Blood test revealed CA 125 antigen level 390.9 (normal = 0-38 U/mL) and rheumatoid factor level 41.6 IU/mL (normal = 0-13.9 IU/mL). Anti–cyclic citrullinated proteins test was not done. Purified protein derivative test was positive with 20 mm; however, 3 sets of sputum for acid-fast bacilli were negative.

Chest X-ray showed atelectasis in lung bases. Abdomen ultrasound computed tomography scan of abdomen and pelvic with intravenous contrast showed large amount of ascites without masses. Transvaginal ultrasound revealed large pelvic ascites and nonvisualization of both ovaries. Diagnostic and therapeutic paracentesis showed yellow, cloudy fluid, white blood cells 910 cells/µL, neutrophils 13 cells/µL, lymphocytes 89 cells/µL, red blood cells 395 cells/µL, total protein 6.1 g/dL, and albumin 2.5 g/dL. Serum-ascites albumin gradient was 0.4.

**Figure 1.**
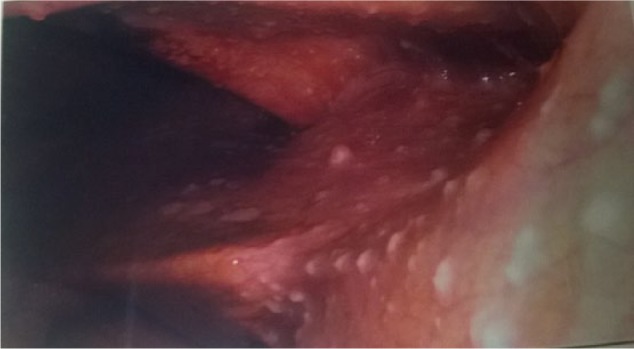
Numerous whitish colored small tuberculous nodules found on peritoneum during laparoscopy.

**Figure 2.**
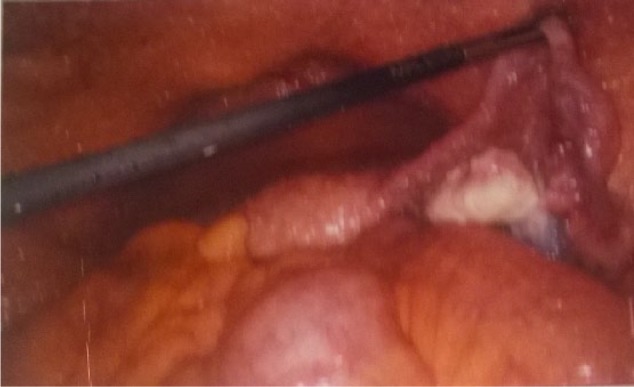
Whitish colored nodules found on peritoneum during laparoscopy.

**Figure 3.**
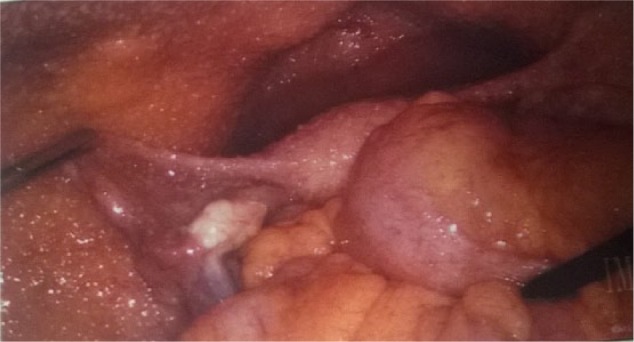
Whitish colored nodules found on peritoneum during laparoscopy.

The patient underwent peritoneal laparoscopy, which showed straw colored ascitic fluid with whitish tuberculous peritoneal nodules (Figures 1-3). Four liters of fluid was drained. Both ovaries were not enlarged and looked grossly normal. Biopsy of peritoneum revealed multiple nonnecrotizing and necrotizing granulomas with Langham type of giant cell and acid-fast bacilli seen on special stain, consistent with mycobacterial infection and negative for malignant cells.

The patient was started on anti-TB drugs (rifampin, isoniazid, pyrazinamide, and ethambutol) with resolution of ascites 2 weeks later.

## Discussion

Although tumor markers are useful for cancer screening, diagnosis, monitoring treatment effectiveness, and recurrence, it should be interpreted appropriately and carefully due to the possibility of elevation in the benign conditions.^4^ CA 125 is a soluble glycoprotein oncofetal antigen that was found in coelomic epithelium during fetal development. CA 125 is widely recognized and utilized in monitoring recurrence of epithelial ovarian cancer, treatment responsiveness, and to distinguish malignant from benign pelvic masses.^4,5^ For premenopausal women, CA 125 levels are usually elevated with benign gynecologic condition such as fibroid, benign ovarian cysts, and endometriosis. Terada et al published a study stating that menopausal females with an elevated CA 125 and without ovarian cancer are associated with increased risk of premature mortality.^6^

Elevation of CA 125 levels may also be associated with benign conditions such as pleuropulmonary diseases, lung infections, chronic liver disease, connective tissue disease, peritoneal dialysis, and recent surgery.^7,8^ Miralles et al published a study of 380 patients who were randomly chosen from a general hospital and 61 patients were found to have high CA 125 levels.^8^ The likely etiologies for these conditions are due to injury or proliferation of mesothelial cells leading to serosal effusions. Because of the similar embryonic developmental origins of pleural and peritoneal lining cells, it is assumed that irritation or inflammation of these areas results in increasing levels of CA 125.^7,8^

Peritoneal TB is a rare condition in Western countries but should be suspected in immigrants or travelers from developing countries. It accounts for 0.1% to 0.7% of all cases of TB.^9^ The main location of pelvic TB is fallopian tubes and symptoms are fever, weight loss, pelvic pain, infertility, and menstrual irregularities. Less common symptoms include ascites, adnexal masses, or combined with an increase level of CA 125. Since it has a variety of clinical manifestation, it is often difficult to distinguish from abdominal malignancies. Diagnosis of peritoneal TB is usually made by histology biopsy features revealing caseating granuloma, positive acid-fast bacillus, culture for mycobacterium TB, or positive polymerase chain reaction.

Tuberculosis infection itself may also stimulate immune system and induce autoimmune reaction, which produces antibodies such as antinuclear antibody, anticardiolipin, antineutrophil cytoplasmic antibodies, rheumatoid factor, and anticyclic citrullinated proteins.^10^ Elkayam et al reported in a study mentioning that 15 out of 47 patients with active pulmonary TB infection had elevation of rheumatoid factor while rheumatology symptoms in these patients were rare.^3^ Another study by Djavad et al found that the rheumatoid factors produced by Epstein-Barr virus transformed monoclonal B cells obtained from patients with rheumatoid arthritis and TB, and revealed that they have similarities in avidity and affinity of different antigens.^3,11^ The elevation of rheumatoid factor in TB patients has significant clinical values because exacerbation of TB infection can be found in patients with rheumatoid arthritis in conditions such as during disease activation and after initiation of biologic agents. High titers of rheumatoid factor are more common in pulmonary TB and tuberculous arthritis compared to peritoneal TB. Our patient has high levels of rheumatoid factor, but did not have any rheumatologic symptoms or disease.

In conclusion, combined elevation of CA 125 and rheumatoid factor levels in one patient with peritoneal TB is a very rare condition in Western countries. A high index of suspicion is required because peritoneal TB is a great mimicker of other abdominal pathology including malignancies and can mislead physicians to undertake unnecessary interventions.
